# Analysis of Hub Genes and the Mechanism of Immune Infiltration in Stanford Type a Aortic Dissection

**DOI:** 10.3389/fcvm.2021.680065

**Published:** 2021-07-02

**Authors:** Haoyu Gao, Xiaogang Sun, Yanxiang Liu, Shenghua Liang, Bowen Zhang, Luchen Wang, Jie Ren

**Affiliations:** Department of Cardiovascular Surgery, State Key Laboratory of Cardiovascular Disease, National Center for Cardiovascular Diseases, Fuwai Hospital, Chinese Academy of Medical Sciences and Peking Union Medical College, Beijing, China

**Keywords:** aortic dissection, immune infiltration, hub gene, bioinformactics, monocyte—macrophage

## Abstract

**Background:** Stanford type A aortic dissection (AAD) is a catastrophic disease. An immune infiltrate has been found within the aortic wall of dissected aortic specimens. The recall and activation of macrophages are key events in the early phases of AAD. Herein, the immune filtration profile of AAD was uncovered.

**Methods:** Gene expression data from the GSE52093, GSE98770 and GSE153434 datasets were downloaded from the Gene Expression Omnibus (GEO). The differentially expressed genes (DEGs) of each dataset were calculated and then integrated. A protein-protein interaction (PPI) network was established with the Search Tool for the Retrieval of Interacting Genes/Proteins (STRING), and the hub genes were identified in Cytoscape. Furthermore, gene ontology (GO) functional annotation and Kyoto Encyclopedia of Genes and Genomes (KEGG) pathway analysis of hub genes were performed. Finally, we set GSE52093 and GSE98770 as the training set and GSE153434 as the validation set to assess immune infiltration in AAD using CIBERSORTx and analyzed the correlations between immune cells and hub genes in both the training and validation sets.

**Results:** Sixty-one integrated DEGs were identified. The top 10 hub genes were selected from the PPI network, and 140 biological process (BP) terms and 12 pathways were enriched among the top 10 hub genes. The proportions of monocytes and macrophages were significantly higher in AAD tissues than in normal tissues. Notably, this result was consistent in the training set and the validation set. In addition, we found that among the hub genes, CA9, CXCL5, GDF15, VEGFA, CCL20, HMOX1, and SPP1 were positively correlated with CD14, a cell marker of monocytes, while CA9, CXCL5, GDF15, and VEGFA were positively correlated with CD68, a cell marker of macrophages in the training set. Finally, according to the results of the GO and KEGG analysis of hub genes, we found that the monocyte/macrophage-related genes were involved in immune-inflammatory responses through degradation of the extracellular matrix, endothelial cell apoptosis, hypoxia and the interaction of cytokines and chemokines.

**Conclusion:** The monocyte-macrophage system plays a major role in immune-inflammatory responses in the development of AAD. Several hub genes are involved in this process via diverse mechanisms.

## Introduction

Aortic dissection is a catastrophic disease characterized by tears in the aortic wall. The intimal tear allows passage of blood into the media, creating a false channel. With each cardiac contraction, the dissected channel can extend proximally or distally, potentially causing rupture as the outer wall weakens. Based on the position of the primary tear and the range of dissection involvement, aortic dissection is classified as Stanford type A (AAD) or Stanford type B. AAD, which is defined as dissection involving the ascending aorta, carries a high risk of mortality and morbidity (1). While type B dissections usually originate distal to the left subclavian artery and do not involve the ascending aorta. Currently, the major pharmacotherapy includes that reducing the blood pressure, controlling the heart rate. No effective drug therapy has been proven to control the development or progression of AAD, and the most valid strategy for AAD is open surgery, which has high technical requirements and must be performed in an experienced aortic center, which is a challenge for medically underdeveloped areas. According to a registry study, the pharmacotherapy rate was 35.6%, the mortality rate was 42.5%, the surgical therapy rate was 52.6%, and the mortality rate was 5.3% for AAD (2). Hence, we need to develop novel pharmacologic therapies and management strategies through in-depth study of the molecular and cellular basis of AAD.

With the development of information technology, bioinformatics analysis can be used as an effective tool to study gene expression profiles and reveal the underlying molecular biological mechanism to provide a direction for future basic research. In recent research, some hub genes and their interactions in AAD were revealed, and underlying pathways involving hub genes were indicated (3–6). In addition, the relationship between AAD and immune-inflammatory mechanisms has been closely examined in some studies (7). Nevertheless, to date, no studies have further assessed immune cell infiltration in aortic dissection and the correlation between immune cells and hub genes through bioinformatics analysis, which may reveal potential therapeutic targets for AAD.

CIBERSORT, a bioinformatics tool, can distinguish and quantify 22 human immune cell types using a deconvolution algorithm based on gene expression data (8). Previous studies have shown that CIBERSORT can be used accurately to evaluate the composition of immune cells in many malignant tumors and non-malignant diseases (9, 10).

In this study, we first downloaded three datasets from the Gene Expression Omnibus (GEO) database and then used R software (version 3.6.1, http://r-project.org/) to standardize all the datasets and screen out differentially expressed genes (DEGs) between AAD patients and non-AAD patients. Subsequently, the RobustRankAggreg (RRA) method was applied to integrate DEGs from each of the three gene profiling datasets. Gene ontology (GO) functional annotation and Kyoto Encyclopedia of Genes and Genomes (KEGG) pathway analysis were performed based on the integrated DEGs. A protein-protein interaction (PPI) network of the integrated DEGs was constructed using the Search Tool for the Retrieval of Interacting Genes/Proteins (STRING) database, and Cytoscape software was used to identify hub genes (11, 12). Moreover, CIBERSORT was utilized to analyze the difference in the infiltration of 22 immune cell subsets between AAD tissues and normal tissues (8). Finally, we further studied the relationship between the hub genes and infiltrating immune cell markers to better understand the underlying mechanism of molecular immunity during the occurrence and development of AAD.

## Materials and Methods

### Data Download

We searched for the datasets including AAD patients in the GEO database (https://www.ncbi.nlm.nih.gov/geo/). The inclusion criteria were as follows: (I) samples in the dataset were collected from the aorta; (II) the samples included AAD patients and healthy controls; (III) the dataset was based on human gene expression profiles. The exclusion criteria included that the raw data of microarray data or the counts data of high-throughput sequencing data were not available. Three datasets, GSE52093, GSE98770 and GSE153434 that met the criteria were included in this study. The platform of the GSE52093 dataset was GPL10558 (Illumina HumanHT-12 V4.0 expression beadchip). The gene expression profiling data of the GSE98770 dataset were obtained via mRNA and miRNA microarrays, and the platform of the mRNA microarrays was GPL14550 (Agilent-028004 SurePrint G3 Human GE 8 × 60K Microarray); these datasets were analyzed in this study. Gene expression profiling data in the GSE153434 dataset were obtained by high-throughput sequencing, and the platform was GPL20795 (HiSeq X Ten).

### Data Processing and DEG Screening

The limma R package (13) was used to correct background, normalize data and screen DEGs from the raw data of GSE52093 and GSE98770 (GPL14550). The DESeq2 R package (14) was used to correct background, normalize data and screen DEGs from the raw data of GSE153434. *P*-values were adjusted using the Benjamini and Hochberg test, and adjusted *p* < 0.05 and |log fold change (FC)| > 1 were considered the cutoff criteria. A boxplot was generated to visualize the effect of processing raw data, and volcano maps and heatmaps of the DEGs were drawn using the ggplot2 R package (https://ggplot2.tidyverse.org) and pheatmap R package (https://CRAN.R-project.org/package=pheatmap) to show the differential expression of each DEG.

### Integration of DEGs

The RRA R package (https://CRAN.R-project.org/package=RobustRankAggreg) was used to integrate the DEGs of three datasets ranked by logFC. The integrated DEGs were visualized with a heatmap.

### GO and KEGG Pathway Enrichment Analyses of Integrated DEGs

The clusterProfiler R package (15) was used to perform the GO and KEGG analysis of integrated DEGs. The results, which were considered statistically significant if the *p* < 0.05, were visualized with a histogram.

### PPI Network Construction

A PPI network of integrated DEGs was established by the STRING online database (version 11.0; http://string-db.org/) (16), and only interacting proteins were selected. Subsequently, we used Cytoscape software (11) to visualize the PPI network and screen for hub genes using the CytoHubba plugin (17). The hub genes were assessed by GO functional annotation analysis and visualized in a network map.

### Analysis of Immune Cell Infiltration

We uploaded the gene expression profiles from GSE52093 and GSE98770 (GPL14550), which are both microarray datasets, to CIBERSORTx (https://cibersortx.stanford.edu/) (8). Then, a bar graph and heatmap were drawn using the ggplot2 R package to visualize the content of immune cell types in each sample according to the results. We filtered out immune cells that were not present in each sample and used the ggpubr R package (https://CRAN.R-project.org/package=ggpubr) to analyze the difference in selected types of immune cells between AAD patients and non-AAD patients with the Wilcoxon test.

### Analysis of the Correlations Between Hub Genes and Immune Cells

We performed Pearson correlation analysis of the hub genes and immune cells to further analyze the immune mechanism during the development of AAD using the ggstatsplot R package (https://CRAN.R-project.org/package=ggstatsplot) and drew a dot plot to show the results.

### Verification of the Immune Infiltration Results

The gene expression profiles of the GSE153434 dataset were uploaded to CIBERSORTx to perform immune infiltration analysis to verify the results of immune infiltration in the GSE52093 and GSE98770 datasets. Then, we used the same correlation test method as above to verify the correlations between DEGs and differential immune cells in the GSE153434 dataset.

## Results

### Data Preprocessing and DEGs Screening

**Figure 1:**
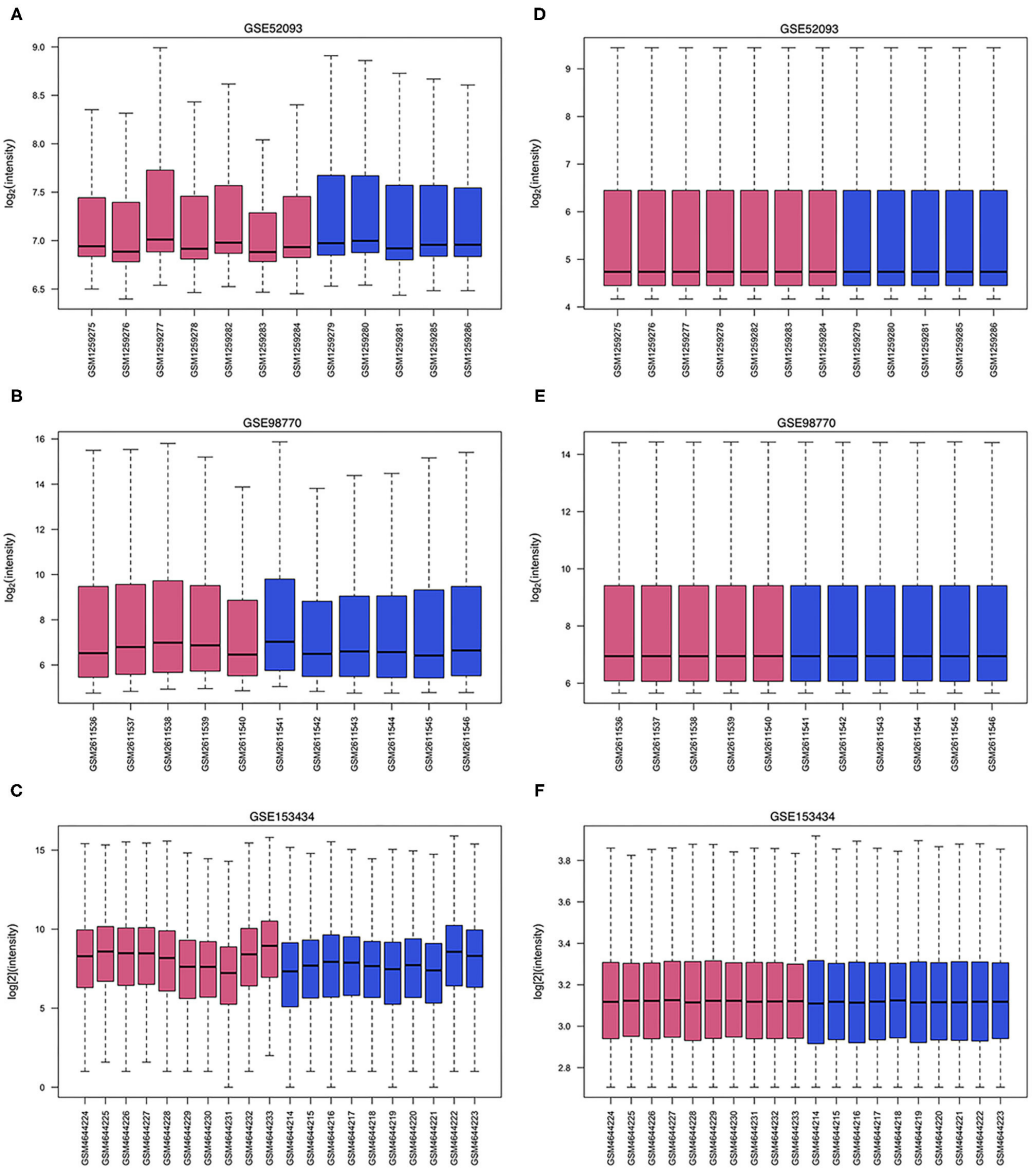
Data processing of gene expression profiles. The red bars represent data from AAD patients, and the blue bars represent data from non-AAD patients. **(A–C)** Raw data of GSE52093, GSE98770 and GSE153434. **(D–F)** Normalized data of GSE52093, GSE98770, and GSE153434.

**Figure 2:**
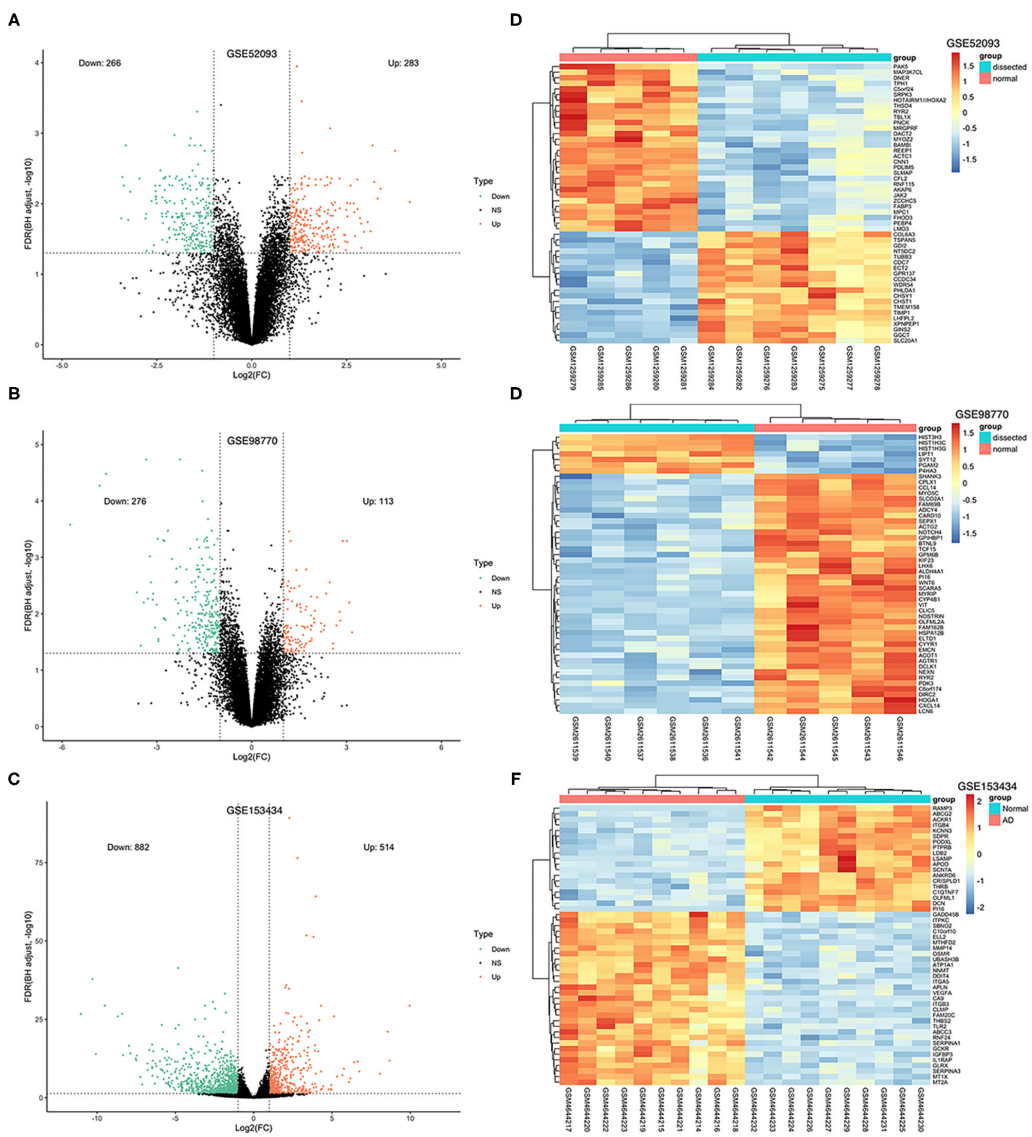
Volcano plots and heatmaps of DEGs from the three datasets. **(A–C)** Volcano plots of DEGs from GSE52093, GSE98770 and GSE153434. The red dots represent upregulated differential genes, the green dots represent differential downregulated genes and the black dots represent genes without significant differences. **(D–F)** Heatmaps of the top 50 DEGs from GSE52093, GSE98770, and GSE153434. Red represents upregulated DEGs, blue represents downregulated DEGS, and the gradation of color represents the value of |log FC|.

The GSE52093 dataset included information on 7 AAD patients and 5 non-AAD patients; the GSE98770 dataset included information on 7 AAD patients and 5 non-AAD patients; and the GSE153434 dataset included information on 10 AAD patients and 10 non-AAD patients. There were not any missing values in three datasets. All data were subjected to normalization and background correction, and the results are shown in Figures 1A–F. Then, based on the cutoff value of |log FC| ≥ 1 and adjusted *p* < 0.05, 549 DEGs, including 283 upregulated genes and 266 downregulated genes, were screened from the GSE52093 dataset; 389 DEGs, including 113 upregulated genes and 276 downregulated genes, were identified in GSE98770; the GSE153434 contained 1396 DEGs, including 514 upregulated genes and 882 downregulated genes. The DEGs of the three datasets are shown in the volcano map (Figures 2A–C), and the top 50 DEGs of the three datasets are shown in the cluster heatmaps (Figures 2D–F).

### Integration of the DEGs Using RRA Analysis

**Figure 3:**
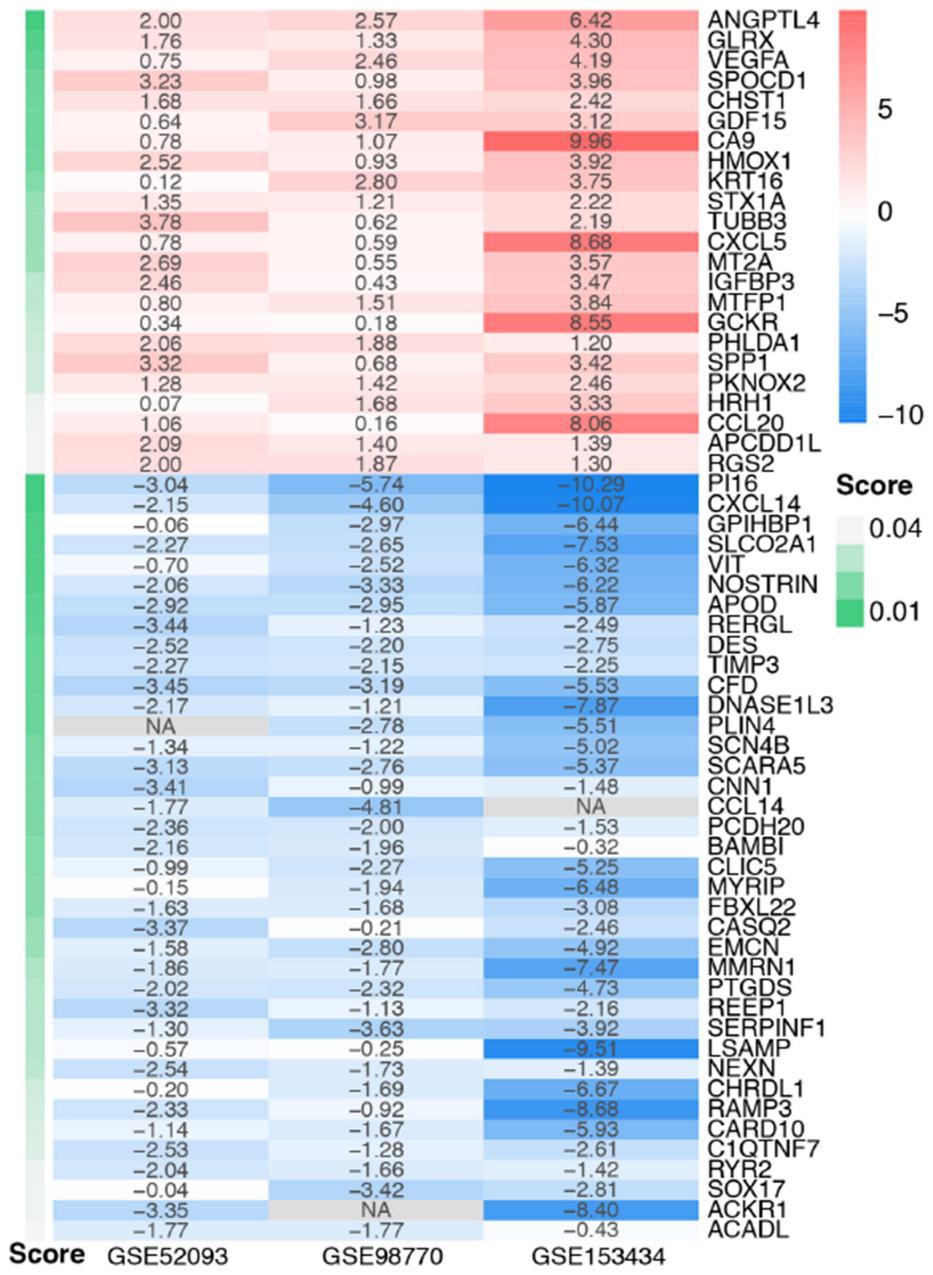
Heatmap of the integrated DEGs. Red represents upregulated genes, blue represents downregulated genes, gray represents genes that were not detected in some datasets, and gradation of green represents the score obtained in the RRA analysis.

The DEGs of the three datasets were sorted according to logFC and integrated using the RRA R package with the cutoff values of adjusted *p* < 0.05 and |log FC| ≥ 1. Through RRA analysis, we obtained 61 integrated DEGs, including 23 upregulated genes and 38 downregulated genes, which are presented in a heatmap in Figure 3.

### GO and KEGG Analysis of Integrated DEGs

**Figure 4:**
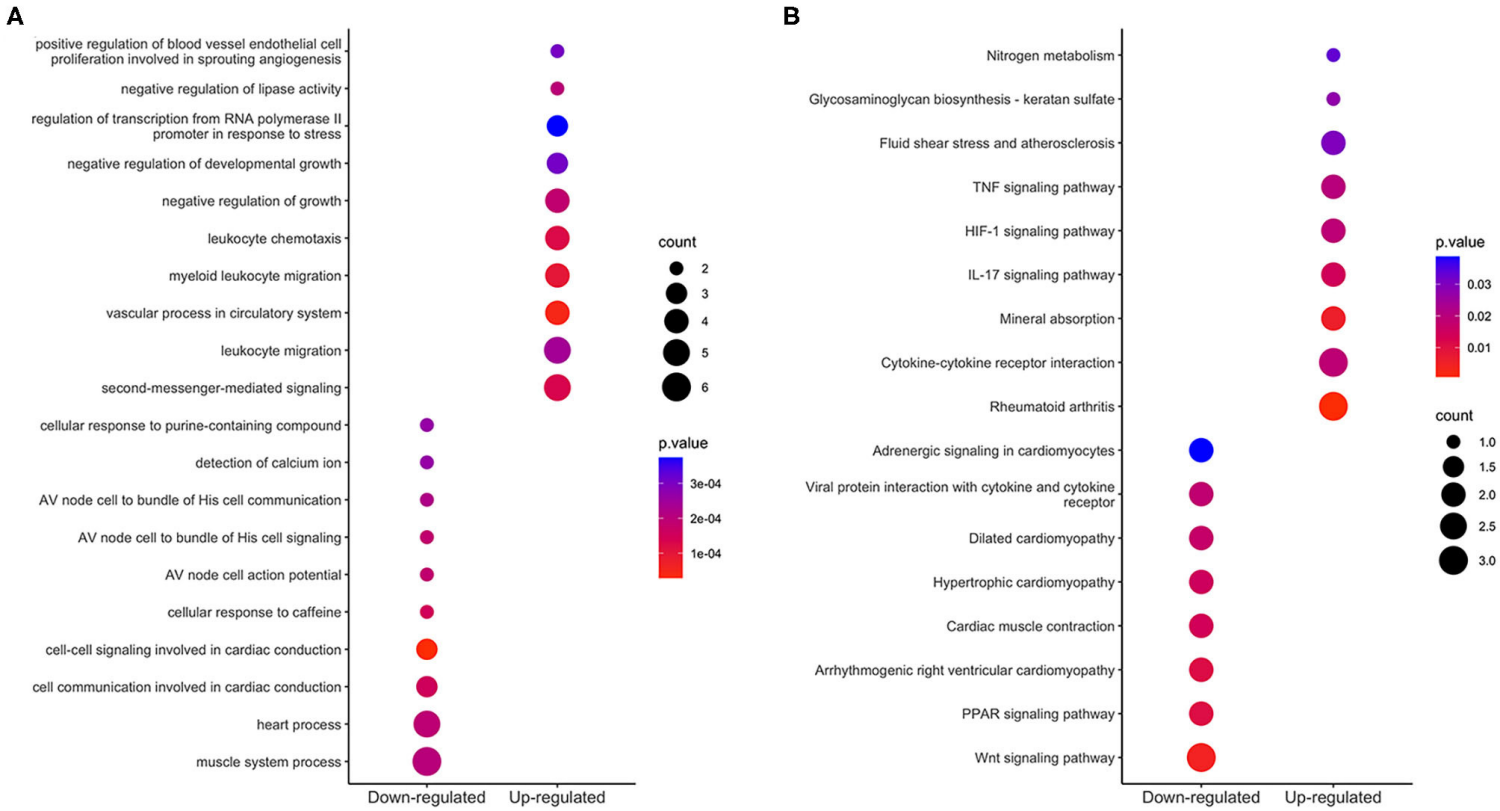
Enrichment analysis of integrated DEGs. The color represents the *p*-value of the terms, while the x axis represents the different gene categories including up-regulated and down-regulated DEGs. Count refers to the number of DEGs enriched in a GO term or KEGG pathway. **(A)** The top 10 terms enriched among up-regulated and down-regulated integrated DEGs. **(B)** The results of KEGG analysis of the upregulated and downregulated integrated DEGs.

We used the clusterProfiler R package to perform GO function annotation and KEGG pathway enrichment analysis. GO function terms were divided into biological process (BP), molecular function (MF) and cell component (CC) categories. We mainly focused on BP terms in this study. According to the cutoff value of *p* < 0.05, 33 BP terms, such as vascular process, leukocyte migration, response to hypoxia and cellular response to cadmium ion, were enriched among the upregulated integrated DEGs; 17 BP terms, such as cardiac conduction, muscle system process, detection of calcium ion, were enriched among the downregulated integrated DEGs. The top 10 BP terms of GO analysis are shown in Figure 4A.

Based on KEGG pathway enrichment analysis, 9 pathways were enriched (with a threshold of *p* < 0.05) among the upregulated integrated DEGs; these pathways included cytokine-cytokine receptor interaction, rheumatoid arthritis, the interleukin-17 (IL-17) signaling pathway, the hypoxia-inducible factor (HIF)-1 signaling pathway and the tumor necrosis factor (TNF) signaling pathway; 8 pathways were enriched among the downregulated integrated DEGs; these pathways included the wnt signaling pathway, PPAR signaling pathway, and heart process-related pathways. The results are shown in Figure 4B.

### PPI Network Construction and Identification of Hub Genes

**Figure 5:**
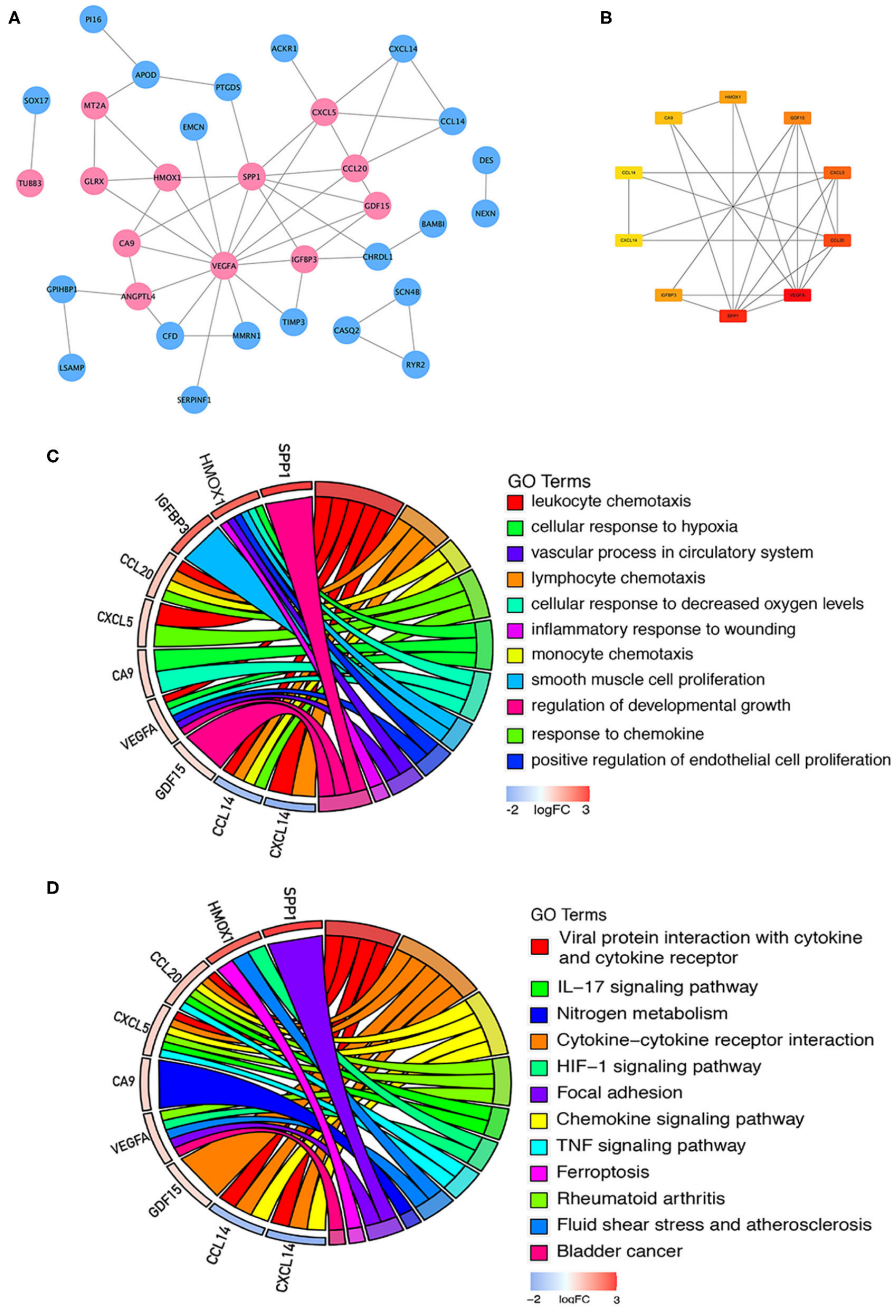
PPI network construction and enrichment analysis of the hub genes. **(A)** PPI network constructed with the integrated DEGs. The red nodes represent upregulated proteins, and the blue nodes represent downregulated proteins. **(B)** Top 10 hub genes calculated by the MCC method in CytoHubba software. The gradation of color represents the value of the score according to the MCC method, and the VEGFA score was the highest. **(C)** GO functional annotation analysis of the top 10 genes. **(D)** KEGG pathway enrichment analysis of the top 10 genes.

Sixty-one integrated DEGs were uploaded to the STRING (https://string-db.org/cgi/input.pl) online database to analyze PPIs, and the interaction data were entered into Cytoscape software to construct a PPI network. The network contains 33 nodes and 51 edges (Figure 5A). Then, we used CytoHubba, a Cytoscape plugin, to analyze the hub genes. According to the method of maximal clique centrality (MCC), the top 10 hub genes included secreted phosphoprotein 1 (SPP1), vascular endothelial growth factor A (VEGFA), C-C motif chemokine ligand 20 (CCL20), growth differentiation factor 15 (GDF15), C-X-C motif chemokine ligand 5 (CXCL5), insulin like growth factor binding protein 3 (IGFBP3), C-X-C motif chemokine ligand 14 (CXCL14), heme oxygenase 1 (HMOX1), carbonic anhydrase 9 (CA9), and C-C motif chemokine ligand 14 (CCL14) (Figure 5B). According to GO function annotation and KEGG pathway enrichment analysis of the top 10 hub genes, 140 BP terms and 12 pathways were enriched, and the results are shown in Figures 5C,D and Table 1.

**Table 1:** GO functional annotation and KEGG pathway enrichment analysis of hub genes.

**Category**	**Term**	**Genes**	***P*-value**
BP	Leukocyte chemotaxis	VEGFA, CCL20, CXCL5, CXCL14, CCL14	0.000
	Lymphocyte chemotaxis	CCL20, CXCL14, CCL14	0.001
	Response to chemokine	CCL20, CXCL5, CCL14	0.001
	Cellular response to hypoxia	VEGFA, HMOX1, CA9	0.006
	Cellular response to decreased oxygen levels	VEGFA, HMOX1, CA9	0.006
	Monocyte chemotaxis	CCL20, CCL14	0.012
	Regulation of developmental growth	VEGFA, SPP1, GDF15	0.014
	Positive regulation of endothelial cell proliferation	VEGFA, HMOX1	0.023
	Smooth muscle cell proliferation	IGFBP3, HMOX1	0.041
	Vascular process in circulatory system	VEGFA, HMOX1	0.042
	Inflammatory response to wounding	HMOX1	0.045
KEGG	Viral protein interaction with cytokine and cytokine receptor	CCL20, CXCL5, CXCL14, CCL14	0.000
	Cytokine-cytokine receptor interaction	CCL20, CXCL5, GDF15, CXCL14, CCL14	0.000
	Chemokine signaling pathway	CCL20, CXCL5, CXCL14, CCL14	0.000
	Rheumatoid arthritis	VEGFA, CCL20, CXCL5	0.000
	IL-17 signaling pathway	CCL20, CXCL5	0.006
	HIF-1 signaling pathway	VEGFA, HMOX1	0.008
	TNF signaling pathway	CCL20, CXCL5	0.008
	Fluid shear stress and atherosclerosis	VEGFA, HMOX1	0.012
	Nitrogen metabolism	CA9	0.021
	Focal adhesion	VEGFA, SPP1	0.024
	Ferroptosis	HMOX1	0.050
	Bladder cancer	VEGFA	0.050

*GO, Gene Ontology; KEGG, Kyoto Encyclopedia of Genes and Genomes*.

### Immune Cell Infiltration Results

**Figure 6:**
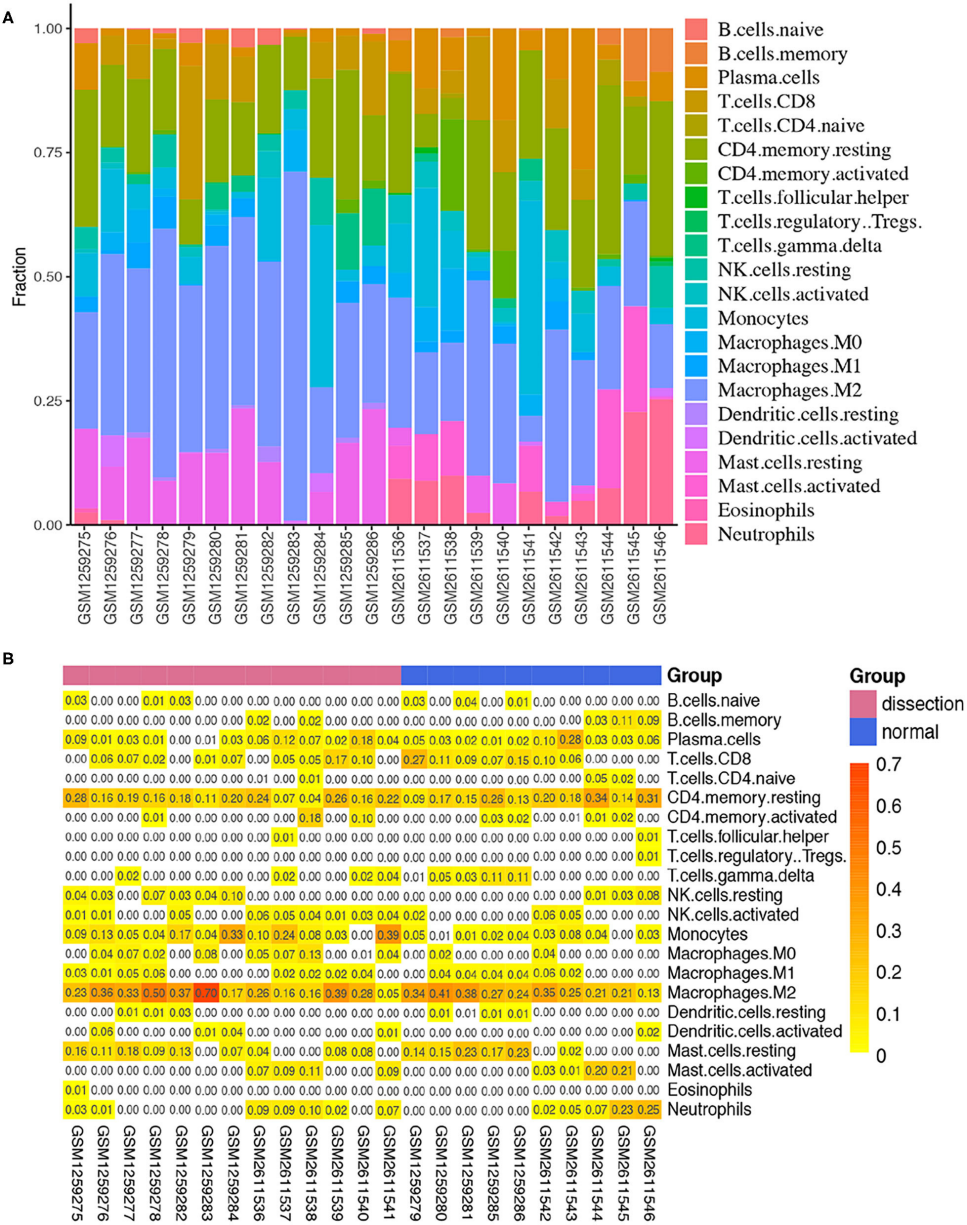
Immune infiltration analysis in the training sets using CIBERSORT. **(A)** Histogram of the fraction of 22 types of immunity in each sample. **(B)** Heatmap of the contents of 22 types of immune cells in each sample.

**Figure 7:**
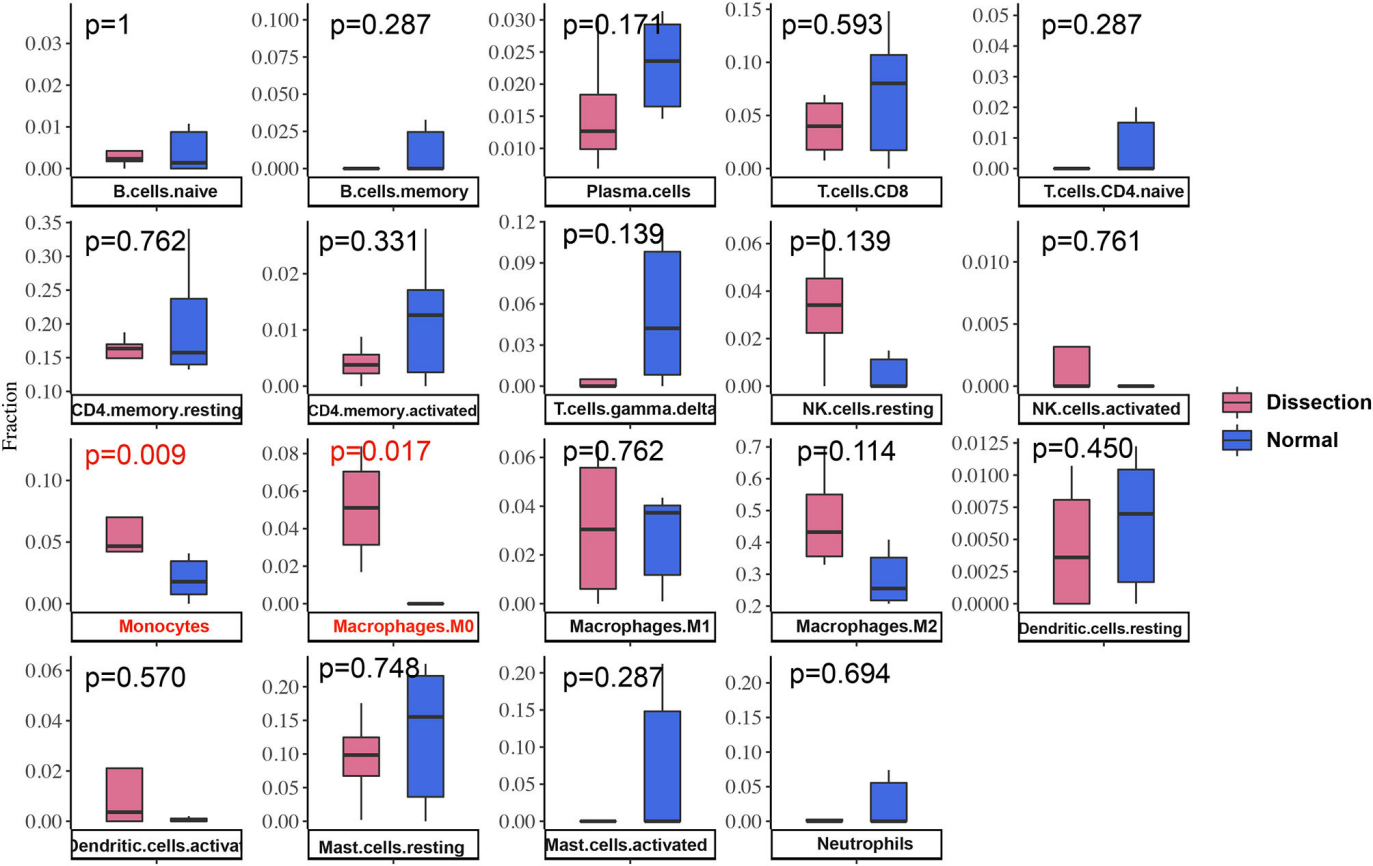
Differences in immune infiltration between AAD samples and non-AAD samples. Red represents AAD samples, and blue represents non-AAD samples. The x axis represents the type of immune cells, and the y axis represents the fraction of immune cells. The Wilcox test was used to analyze differences in immune infiltration. The fraction of monocytes and macrophages. The proportion of M0 macrophages was significantly higher in AAD samples than in non-AAD samples.

After normalization and background correction, the GSE52903 and GSE98770 (GPL10558) datasets were uploaded to CIBERSORTx to analyze immune cell infiltration. CIBERSORTx calculates a *p*-value of the deconvolution of each sample, which provides a measure of the confidence in the results, and a *p* < 0.1 was considered reliable in this study (Figure 6). We filtered out the immune cell types that were not present in the samples, namely, follicular helper T cells and regulatory T cells (Tregs). The remaining 20 types of immune cells were subjected to differential infiltration analysis, and the results indicated that the fractions of monocytes and macrophages between AAD patients and non-AAD patients were significantly different (*p* < 0.05) (Figure 7).

### Analysis of the Correlations Between Hub Genes and Immune Cells

**Figure 8:**
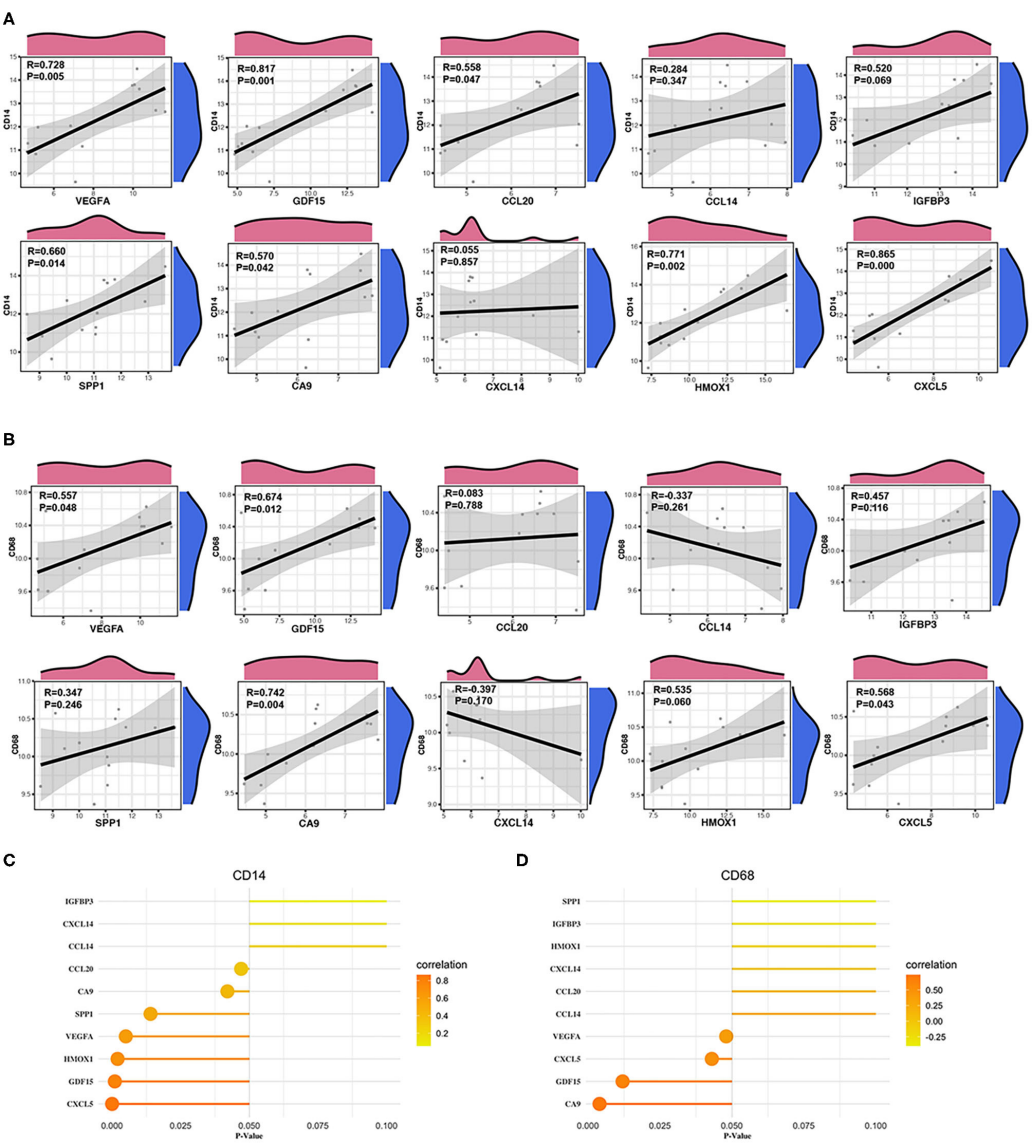
Correlation between monocytes, macrophages and hub genes. **(A)** Scatter diagrams from the correlation analysis. The x axis represents the top genes, and the y axis represents CD14, which is a cell marker of monocytes. **(B)** Scatter diagrams from the correlation analysis. The x axis represents the top genes, and the y axis represents CD68, which is a cell marker of macrophages. **(C,D)** The x axis represents the *P*-value, and the color gradation represents the correlation coefficient. Pearson's test was used for correlation analysis.

We calculated the Pearson correlations between the hub genes and CD14 and CD68, which are cell markers of monocytes and macrophages, respectively, based on the CellMarker database (http://bio-bigdata.hrbmu.edu.cn/CellMarker/). The results indicated that CA9 (correlation coefficient = 0.570, *p* = 0.042), CXCL5 (correlation coefficient = 0.865, *p* = 0), GDF15 (correlation coefficient = 0.817, *p* = 0.001), VEGFA (correlation coefficient = 0.728, *p* = 0.005), CCL20 (correlation coefficient = 0.558, *p* = 0.047), HMOX1 (correlation coefficient = 0.771, *p* = 0.002), and SPP1 (correlation coefficient = 0.660, *p* = 0.014) were positively correlated with CD14 (corresponding to monocytes); CA9 (correlation coefficient = 0.742, *p* = 0.004), CXCL5 (correlation coefficient = 0.568, *p* = 0.043), GDF15 (correlation coefficient = 0.674, *p* = 0.012), and VEGFA (correlation coefficient = 0.557, *p* = 0.048) were positively correlated with CD68 (corresponding to macrophages) (Figures 8A–D, Table 2). In addition, we also chose CD86 as the marker of M1 macrophage and CD163 as the marker of M2 macrophage to perform correlation analysis as supplement and the results were shown in Table 3. GO function annotation and KEGG pathway enrichment analysis of the top 10 hub genes revealed that monocyte/macrophage-related genes were involved in immune-inflammatory responses dominated by monocytes and macrophages through degradation of the extracellular matrix, endothelial cell apoptosis, hypoxia and the HIF-1 signaling pathway and the interaction of cytokines and chemokines.

**Table 2:** Correlation analysis between immune cell and hub genes in the training set and the validation set.

**Cell marker**	**Gene**	**Training set**		**Validation set**	
		**Correlation coefficient**	***P*-value**	**Correlation coefficient**	***P-*value**
CD14	CA9	0.570	0.042	0.658	0.039
	CCL14	0.284	0.347	–	–
	CCL20	0.558	0.047	0.122	0.736
	CXCL14	0.055	0.857	–	–
	CXCL5	0.865	0	0.399	0.253
	GDF15	0.817	0.001	0.803	0.005
	HMOX1	0.771	0.002	0.765	0.010
	IGFBP3	0.520	0.069	–	–
	SPP1	0.660	0.014	0.256	0.474
	VEGFA	0.728	0.005	0.312	0.381
CD68	CA9	0.742	0.004	0.683	0.03
	CCL14	−0.337	0.261	–	–
	CCL20	0.083	0.788	–	–
	CXCL14	−0.397	0.179	–	–
	CXCL5	0.568	0.043	0.427	0.219
	GDF15	0.674	0.012	0.667	0.035
	HMOX1	0.535	0.06	–	–
	IGFBP3	0.457	0.116	–	–
	SPP1	0.347	0.246	–	–
	VEGFA	0.557	0.048	0.383	0.274

*CD14, cell marker of monocyte; CD68, cell marker of macrophage; Pearson test was used to perform correlation analysis*.

**Table 3:** Correlation analysis between macrophage M1/M2 and hub genes in the training set and the validation set.

**Cell marker**	**Gene**	**Training set**		**Validation set**	
		**Correlation coefficient**	***P*-value**	**Correlation coefficient**	***P*-value**
CD86	CA9	0.770	0.002	−0.030	0.935
	CCL14	0.167	0.586	−	-
	CCL20	0.429	0.143	−	-
	CXCL14	−0.032	0.917	−	-
	CXCL5	0.839	0	0.667	0.035
	GDF15	0.861	0	−0.397	0.046
	HMOX1	0.752	0.003	0.145	0.690
	IGFBP3	0.519	0.069	−	-
	SPP1	0.559	0.047	0.878	0.001
	VEGFA	0.997	0.000	−0.200	0.580
CD163	CA9	0.604	0.029	−0.217	0.546
	CCL14	0.279	0.355	−	-
	CCL20	0.654	0.015	−0.412	0.236
	CXCL14	0.011	0.973	−	-
	CXCL5	0.864	0	0.480	0.010
	GDF15	0.819	0.001	−0.591	0.072
	HMOX1	0.834	0	−0.222	0.008
	IGFBP3	0.585	0.036	0.501	0.097
	SPP1	0.715	0.006	0.852	0.002
	VEGFA	0.769	0.002	−0.579	0.008

*CD86, cell marker of M1 macrophage; CD163, cell marker of M2 macrophage; Pearson test was used to perform correlation analysis*.

### Verification of the Immune Infiltration Results

**Figure 9:**
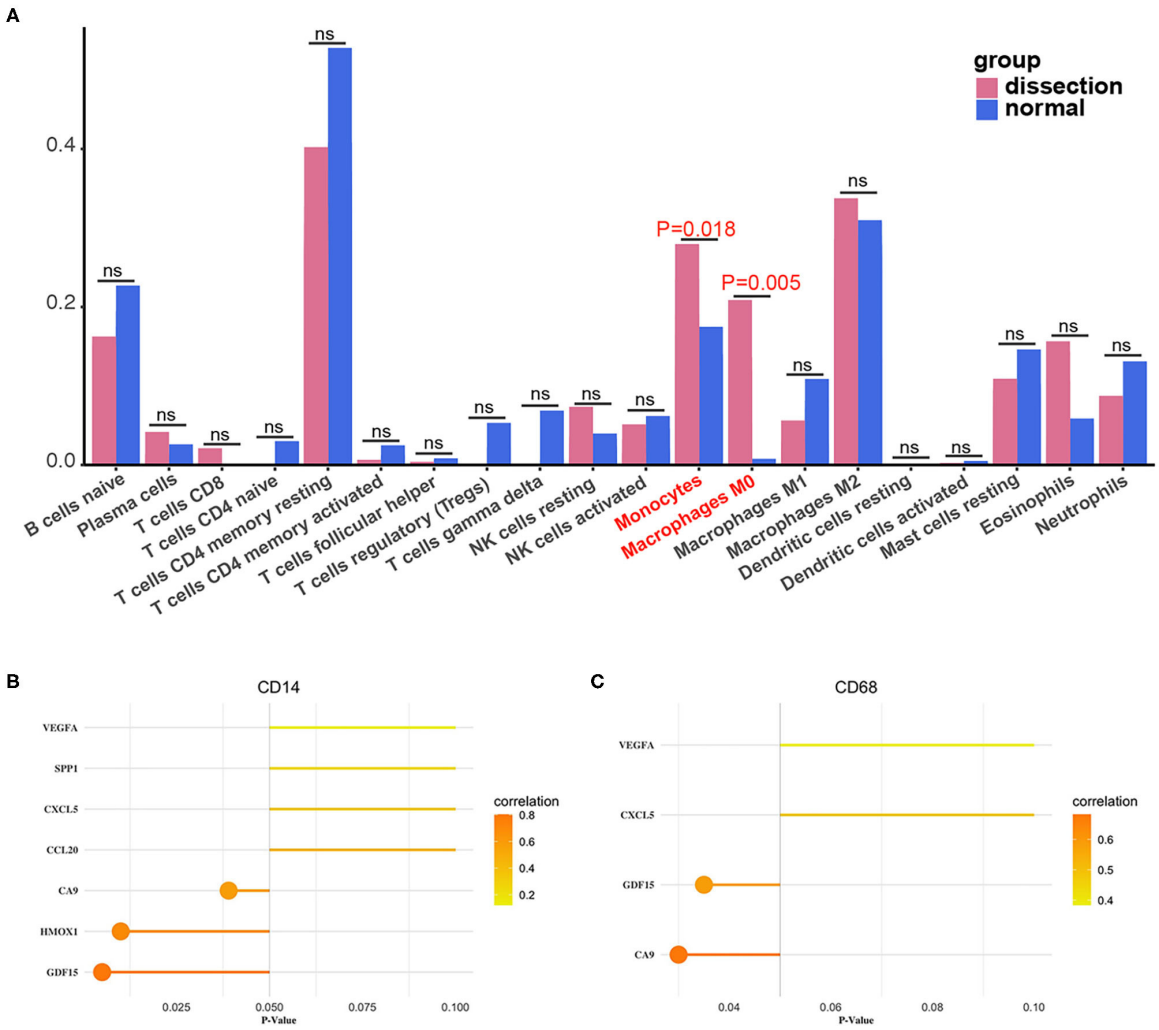
Validation of immune infiltration and correlation analysis. **(A)** The histogram of the fraction of immune cells in the validation set. Red indicates the AAD samples, and blue indicates the non-AAD samples. The Wilcoxon test was performed, and the results showed a significant difference in the fraction of immune cells between AAD samples and non-AAD samples, which was in accordance with results from the training set. **(B,C)** Analysis of the correlation between immune cells and hub genes in the validation set with Pearson test.

The fractions of monocytes and macrophages were also significantly different between AAD patients and non-AAD patients in GSE153434 according to immune infiltration analysis with CIBERSORTx (Figure 9A). We also verified the results of the correlation analysis in the training set using the GSE153434 dataset. In the validation set, the results of the correlation analysis were partially consistent with the results in the training set (Figures 9B,C).

## Discussion

Aortic dissection is a life-threatening disease with a high mortality rate that is characterized by separation of the aortic wall caused by intima injuries that result in blood flow into the aortic wall through the intimal tear. According to a study in the International Registry of Acute Aortic Dissection (IRAD), the mortality of AAD patients within 48 h of onset is close to 2% per hour without treatment, and the 3-month mortality rate is 90%. With pharmacotherapy such as β-blockers, the mortality rate within 24 h of AAD is close to 20%, and the 48-h mortality rate is ~30% (1). With surgical treatment, the mortality rate of AAD within an hour is 10%, and the 1-month mortality rate is ~20% (1, 18). It is clear that for AAD, the efficacy of traditional pharmacotherapy is limited, while surgical treatment is very difficult and technically demanding and has a high mortality rate. Hence, it is necessary to find a novel, more effective and less invasive treatment strategy.

Microarray and high-throughput sequencing are powerful and effective technologies to research and predict underlying molecular and cell mechanisms and responses to inspire new therapeutic ideas for patients with AAD (19). Several previous studies have studied hub genes of AAD and their function in the development of AAD to provide new insight for AAD with the aid of microarray and high-throughput sequencing technologies. However, these studies only identified hub genes and did not further analyze the underlying molecular mechanisms in the occurrence and development of AAD or the role of hub genes in certain mechanisms (3–6). In this study, we not only analyzed the hub genes of AAD but also innovatively studied immune infiltration in AAD. In addition, the correlation between markers of immune cells and hub genes was analyzed to further investigate the pathological mechanisms.

First, we used RRA analysis to integrate the three AAD datasets from the GEO database rather than simply overlapping the results. Traditionally, DEGs from different datasets are identified and then combined, which may ignore some genes that have important functions, while the differences in statistics are not significant in certain datasets. In this study, we used the RRA method, which can integrate different gene expression data by ranking genes by their fold changes. Final comprehensive and rigorous results were then achieved by aggregating these rankings (20).

In total, 61 integrated DEGs were identified, which included 23 upregulated genes and 38 downregulated genes. The results of KEGG pathway analysis showed enriched of the HIF-1 signaling pathway, which is related to the immune response of AD and will be discussed in later sections (21). In the subsequent analysis of GO functional enrichment, we focused on the BP group, and the results showed that the upregulated DEGs were mainly enriched in vascular processes, responses to stress, immunoinflammatory responses and positive regulation of blood vessel endothelial cell proliferation. The downregulated DEGs were mainly enriched in heart and muscle system processes and the regulation of calcium ions. Through construction of a PPI network and analysis with the CytoHubba plugin in Cytoscape software, the top 10 hub genes, namely, VEGFA, SPP1, CCL20, CXCL5, GDF15, IGFBP3, HMOX1, CA9, CXCL14, and CCL14, were filtered. Then, analysis of the immune infiltration of AAD was performed with the aid of CIBERSORTx.

The current standard method for assessing the infiltration of immune cells in tissues is immunohistochemistry. However, quantifying and comparing different cell subpopulations is still challenging. In addition, multiple parameters are needed for accurate immunophenotyping. Hence, immune infiltration assessment by immunohistochemistry still needs to be improved. Another method for quantificationally assessing immune infiltrates with multiple parameters is flow cytometry, but this approach cannot be used with limited sample amounts. In addition, choosing the right antibody and optimizing working conditions are crucial for both methods. In comparison, CIBERSORTx analysis requires a small amount of sample without relying on surface markers. Previous studies have reported that CIBERSORTx can accurately evaluate the profile of tumor infiltrating immune cells (9, 22). Badve et al. reported integration of mutations with CIBERSORT analysis could provide better prediction of outcomes and novel therapeutic targets in Triple Negative Breast Cancer (23). Kawada et al. indicated that Infiltrating immune cell subsets detected by CIBERSORT analysis can reflect the time course of innate and adaptive immune responses in acute myocarditis and suggested that CIBERSORT is a promising tool to analyze immune cell landscapes and provide novel insights into the pathogenesis of acute myocarditis (10).

Because microarray data were suggested for immune infiltration analysis via CIBERSORTx in previous studies, we set the microarray gene expression profiles of GSE52093 and GSE98770 as the training set and the high-throughput sequencing data of GSE153434 as the validation set to validate the reliability of the results of the training set (8). Notably, compared with that in non-AAD tissues, the fraction of monocytes and macrophages in AAD tissues was significantly increased in both the training set and validation set, which indicates that the results were reliable. Analysis of the correlations between hub genes and CD14 and CD68, cell markers of monocytes and macrophages, in both the training set and validation set was performed to investigate the mechanism of immunoinflammatory responses in the development of AAD (7, 21, 24).

In our study, less infiltrated neutrophils were found in dissected aorta samples than in normal aorta samples. Although previous studies have reported the infiltration of neutrophils in the aortic wall of AAD patients. For example, Okada et al. indicated that AAD was initiated by neutrophils infiltration in the aortic intima and release of matrix metalloproteinase (MMP) in response to angiotensin II (25). Moreover, Sano et al. reported that massive neutrophils were accumulated in the dissected aorta due to systemic changes in chemokine-dependent signaling induced by the mechanical injury and stretching (26). Neutrophil infiltration is more likely to be predominant in the acute phase of human AAD (27). Burke et al. indicated that adventitial neutrophils occurred in the first 12 h, peaked between 12 and 24 h, and decreased 2 days after the rupture of the false lumen. Unlike neutrophils, macrophages presented 1 day and peaked between 2 and 7 days (28). Although the interval between the onset of aortic dissection and the surgery varies from each patient, the operation is likely to be performed 12–24 h after the onset of aortic dissection in some patients based on our experience. Because some hospitals do not have the conditions to perform this operation and the symptoms of aortic dissection are likely to be confused with angina pectoris. It is likely that the dissected aorta samples were not collected during the acute phase, therefore the infiltrated neutrophils in the aortic walls started to decrease and macrophages began to increase. In future studies, we recommend recording the time of symptom appearance in detail and analyze the immune infiltration according to the group of different immune status.

Arterial wall remodeling is regulated by the immune response and participates in the complicated interaction between cells and immune-inflammatory factors (29). Previous studies have shown that macrophages and their related products play an essential role in the activation and maintenance of inflammation and matrix degradation in AAD (30–32). Macrophages increase the content of matrix metalloproteinases (MMPs) in the arterial wall by upregulating the expression of MMP-2, thereby directly degrading the elastic fibers and extracellular matrix of the arterial wall (33). Then, the degradation of elastic fibers can further facilitate the aggregation of monocyte-macrophages (MMs), which promote the expression of MMPs, resulting in a positive feedback loop. Moreover, we also analyzed the correlation between hub genes and M1/M2 macrophages, which were important in inflammatory response. M1 macrophages are proinflammatory through their release of proinflammatory cytokines, such as TNF-α and IL-1β. TNF-α. While M2 macrophages are considered anti-inflammatory, aiding in the healing process by release of IL-10 and profibrotic factors such as TGF-β (34). Baxter et al. reported that injection of M2 polarized macrophages in mice reduced aortic dilation after aneurysm induction via promoting an anti-inflammatory environment in aortic tissue (35). Hence, altering the M1/M2 ratio may play an important role in slowing or preventing AAD rupture.

The dysfunction of vascular smooth muscle cells (VSMCs) has been shown to have an important role in arterial wall remodeling and the development of AAD in previous studies (36–39). An increase in intracellular calcium concentration in VSMCs triggers the contractile response and leads to VSM fiber shortening and force generation (40, 41). Macrophage-derived cytokines also upregulate the expression of Fas-associated proteins in VSMCs through complex cell interactions, which initiate apoptotic programs and reduce VSMCs, thereby destroying the integrity of the arterial wall (42). A recent study showed that upregulated expression of Fas-associated protein with death domain (FADD) and activation of the apoptosis pathway led to downregulation of miR-27a and induction of apoptosis in endothelial cells (ECs). Then, aortic dissection was induced through promotion of VSMC migration by ECs after downregulation of miR-27a (43).

MMs are essential in the innate immune system and for triggering further immune responses in the occurrence and development of aortic dissection. Normally, the inflammatory response and hypoxia reaction are mutually dependent (7). HIFs, especially HIF-1, are activated in response to the hypoxic and inflammatory microenvironment (44). One study indicated that macrophages induced HIF-1 activation in the development of aortic dissection through distinct metabolic reprogramming, especially fumarate accumulation. Activated macrophage HIF-1 increases disintegrin and metallopeptidase domain 17 (ADAM17) to trigger the inflammatory response, extracellular matrix degradation and elastic plate breakage (21). Furthermore, activated macrophage HIF-1 binds the promoters of CA9, VEGFA and HMOX1, recruits p300 and CBP, and enhances their expression (45, 46).

Cytokines and chemokines have been shown to be involved in aortic dissection by participating in the immune-inflammatory response (47). Of these cytokines, IL-17 was shown to be critically related to AAD. Previous studies reported that circulating IL-17 levels were elevated in patients with AAD (48, 49). Zhang et al. demonstrated that IL-17 induced MMs infiltration by increasing the levels of monocyte chemotactic protein (MCP) 1, MCP2 and MCP3 (50).

In this study, MMs were indicated to play an essential role in the immune infiltration of AAD. Several mechanisms, including those involving degradation of the extracellular matrix, endothelial cell apoptosis, the hypoxia response, the HIF-1 signaling pathway, the interaction of cytokines and chemokines and other pathways, were found to induce the infiltration of MMs and play an important role in the development of AAD. Furthermore, correlation analysis showed that CA9, CXCL5, GDF15, VEGFA, CCL20, HMOX1, and SPP1 were associated with infiltration of MMs. According to GO and KEGG analyses, we analyzed the underlying mechanism of the abovementioned genes involved in the immune response.

IL-17A and IL-17F upregulation increased the levels of CXCL5 and CCL20 through NF-κB and MAPK signaling pathways (51, 52). C-C motif chemokine receptor 6 (CCR6), which is a receptor for CCL20, binds to CCL20 and subsequently triggers calcium-mediated cell transduction (53). CCL20-CCR6 plays an important role in the chemotaxis of dendritic cells (DCs), effector/memory T cells and B cells and the inflammatory response initiated by tissue trauma (54). In addition, IL-17E upregulation activates the apoptosis signaling pathway and reduce VSMC abundance, thereby destroying the integrity of the arterial wall (51, 52). In the HIF-1 signaling pathway, activated macrophage HIF-1 enhances the expression of CA9, VEGFA and HMOX1. VEGFA-mediated angiogenesis and matrix degradation also play a role in AAD (32). Moreover, GDF15 is reported to contain p53 transcription factor binding sites, and the activation of p53 is indispensable in the response to inflammation, oxidative stress and hypoxia (55, 56). Furthermore, GDF15 blocks the synthesis of TNF and nitric oxide (NO) by inhibiting NF-κB signal transduction of and suppresses the activity of macrophages (57). However, the mechanism of GDF15 in the occurrence and development of aortic dissection needs to be confirmed in further research.

Currently, some studies have investigated novel treatment options based on the inflammatory response in aortic dissection. HOMX1 has been demonstrated to be a protective factor in cardiovascular health, and excessive vascular inflammation has been observed in HOMX1-deficient humans (58–60). Ho et al. investigated the role of HOMX-1 in angiotensin II-induced AAA formation in HO-1+/+apoE-/- and HO-1-/-apoE-/- mice and demonstrated the essential roles of HOMX-1 in suppressing the pathogenesis of abdominal aortic aneurysm (61). In addition, Ohno-Urabe et al. indicated that Socs3 in macrophages worked as a protective factor in AAD by preventing excessive inflammation and promoting the tissue repair response, including proper modulation of VSMC function (62). A study showed that CD31 signaling promotes the healing of dissected aorta in mice by switching macrophages from M1 to M2 (63). Indomethacin was demonstrated to reduce the rate of onset of aortic dissection in a murine model by decreasing monocyte/macrophage accumulation in the aortic wall and reducing monocytic transendothelial migration activity (64). Son et al. reported that local and systemic anti-inflammatory responses were activated through enriching anti-inflammatory M2 macrophages in the spleen and peripheral blood at early phase of aortic injury, which finally prevented the development of AAD in animal model (65). To date, pharmacotherapy for AAD is mainly focused on reducing blood pressure and controlling heart rate. However, more novel perspectives on molecular targeted therapy for AAD will be revealed with the in-depth study on the mechanism of immune inflammatory response of AAD.

## Limitations

First, this study needed further examination to determine the results *in vivo*. To improve the reliability of the results, we used two datasets as the training set and the other dataset as the validation set. Additionally, the original sample size was somewhat small, so three datasets were selected due to the low incidence of AAD. Finally, the raw data lacked corresponding clinical information, which may reveal new research perspectives when combined with our results.

## Conclusion

In summary, we demonstrated that MMs plays important roles in immune infiltration in the development of AAD and discussed their underlying mechanisms. CA9, CXCL5, GDF15, VEGFA, CCL20, HMOX1, and SPP1 are involved in this BP through diverse mechanisms, such as the degradation of the extracellular matrix, endothelial cell apoptosis, the hypoxia response, the HIF-1 signaling pathway, and the interaction of cytokines and chemokines, which may reveal a novel perspective on therapeutic targets for AAD.

## Data Availability Statement

The datasets presented in this study can be found in online repositories. The names of the repository/repositories and accession number(s) can be found at: https://www.ncbi.nlm.nih.gov/geo/, GSE52093; https://www.ncbi.nlm.nih.gov/geo/, GSE98770; https://www.ncbi.nlm.nih.gov/geo/, GSE153434.

## Ethics Statement

The studies involving human participants were reviewed and approved by Fuwai Hospital, Chinese Academy of Medical Sciences, and Peking Union Medical College. Written informed consent for participation was not required for this study in accordance with the national legislation and the institutional requirements.

## Author Contributions

XS and HG conceived and designed this study. HG, YL, and SL analyzed data, prepared figures, as well as prepared, and edited the manuscript. HG, JR, and LW performed bioinformatic analyses. XS, BZ, and HG wrote and revised manuscript. All authors reviewed the final manuscript.

## Conflict of Interest

The authors declare that the research was conducted in the absence of any commercial or financial relationships that could be construed as a potential conflict of interest.
